# Spinal Anesthesia from the Standpoint of the Patient

**Published:** 1908-04-15

**Authors:** John S. Marshall

**Affiliations:** Examining and Supervising Dental Surgeon, U. S. Army


### SPINAL ANESTHESIA FROM THE STANDPOINT OF THE PATIENT.

BY JOHN S. MARSHALL, M.D.
Examining and Supervising Dental Surgeon, U. S. Army.

Read before the San Francisco Dental Association, October, 1907.

In relating my personal experience as a patient with spinal anesthesia, I do so with the hope of adding something to the knowledge of the physiological effects of cocain when used in this manner for producing insensibility to pain in surgical operations.

Spinal anesthesia has at the present time but few advocates in the profession, and will, I think, for obvious reasons, never become popular with the public; nevertheless, it is a safe and valuable mode of producing anesthesia in certain cases, and should, therefore, be accorded the recognition which its successful use in hundreds of cases entitles it to receive at the hands of the profession.

After witnessing several operations made upon various portions of the body by Dr. Morton, of San Francisco, in which this method of anesthesia was used with apparent success, I became interested from the standpoint of the oral surgeon, and determined to try it myself upon the first suitable case presenting that required operation upon the maxillary bones. After several months a patient presented who had a fracture of the mandible that required extensive wiring, and with the consent of the patient this method was tried. It, however, proved a signal failure, as the patient declared there was no insensibility to pain nor the slightest anesthesia in the upper extremities, neck or head.

Maj. J. M. Kennedy, surgeon U. S. A., who assisted me at this operation, had similar experiences in other cases in which he had used this method of anesthesia. He, however, was generally successful in producing complete anesthesia in those portions of the body that are supplied by the sacral plexus, and the only unpleasant symptom encountered was a persistent headache, continuing sometimes for two or three weeks after the injection.

My interest in this method of producing anesthesia was so great that I determined, should it become necessary for me to submit to a surgical operation, to insist upon the use of spinal anesthesia, that I might have the opportunity of studying its effects from the personal and practical standpoints and thus settle in my own mind at least its merits and disadvantages.

On January 11, 1907, it became necessary for me to enter the U. S. Army General Hospital, Presidio, San Francisco, for an operation for double inguinal hernia.

The disability was of long standing, having been incurred in a railroad accident in 1864, while en route to the front with my regiment. I am at this date sixty years of age; heart, lungs and kidneys in a normal condition, and in all other respects in perfect health and condition. The operations were performed by Major Kennedy, assisted by Captain Shaw and Lieutenant O'Connor, assistant surgeons, U. S. A.

The patient after being prepared for the operation is seated upon the operating table and told to lean forward and arch the back as much as possible. This seperates the vertebrae, dorsally, to the fullest extent. The lumbar region is then thoroughly scrubbed with soap and hot water, washed with bichloride solution 1 to 1000, followed with alcohol. A hypodermic syringe, glass barrel, with piston down, which has been previously sterilized and charged with one grain of sterilized tropa-cocain (the usual dose), is now handed to the surgeon and the needle inserted between the third and fourth lumbar vertebrae and carried forward until it enters the spinal canal; the piston is then gradually withdrawn until the barrel is filled with the spinal fluid, which is allowed to remain until the cocain is dissolved. This takes but a few seconds, and the piston is then as gradually carried to its former position, returning the fluid charged with the cocain to the spinal canal. The needle is then withdrawn, the puncture washed with alcohol and sealed with collodion.

The introduction of the needle is no more painful than for an ordinary hypodermic injection. Upon withdrawing the spinal fluid from a canal a slightly painful sensation, like that from heavy pressure, was experienced. This was doubtless due to the establishment of a partial vacuum within the canal, as the pain immediately passed off upon the return of the spinal fluid to the canal. As soon as the needle puncture was dressed I was laid upon the operating table and the area of the operations again cleansed.

In about one minute after the cocain was injected a sensation of numbness was experienced in the toes; at the end of two minutes this sensation had extended to the knees; and in three minutes it had reached the umbilicus. Accompanying the numbness in the legs was a sensation of great weight. It seemed as though they weighed tons, and that by the greatest effort it would be impossible to move them, yet upon making the attempt to move the toes and flex the legs at the knee it was found that motion was not impaired.

The operation upon the right side was now begun, and the tissues proved to be completely anesthetic. While the operation was in progress I was studying the further effects of the cocain upon other portions of the body.

In five minutes after the injection, numbness was experienced in the fingers and hands. Sensation was entirely suspended in the third and fourth fingers of both hands, but anesthesia was not complete in the thumb nor in the first and second fingers. No sensation of weight was experienced in either of the hands or arms, and although a slight numbness was experienced from the shoulders downward to the hands, there was very little diminution of sensation in the shoulders, or in the region of the brachial plexus. The neck and head were in no wise affected by the drug, sensation remaining complete and unimpaired in these portions of the body. In about twenty minutes after the introduction of the cocain sensation was completely restored in the fingers, hands and arms.

The operation upon the right side was unusually prolonged on account of the complicated nature of the hernia, viz., direct and oblique combined, and numerous adhesions of the hernial sac with the walls of the inguinal canal. The time consumed in this operation was just thirty minutes. No sensation of pain was experienced during this operation until the introduction of the first superficial suture. It was very evident from the increased pain caused by the introduction of the other three sutures that the anesthetic effect of the drug was rapidly passing off and that in all probability that operation upon the left side would be somewhat painful. I was, however, willing to endure this rather than to take a second injection, or to postpone the second operation to a further date, as I was anxious to have the whole surgical procedure completed at that time, and, furthermore, I desired to carry out my study of the effects of the drug to the end. I therefore requested the surgeon to go on with the second operation, assuring him that I could endure it, and that I would not flinch under the knife.

I nearly repented of this, however, in the next few minutes for the first touch of the knife was quite painful, and gradually increased in severity as the operation progressed.

The most painful part of this operation was the tying of and amputating the hernial sac. At this time I experienced a nauseating and sinking sensation in the region of the solar plexus and for a few moments I feared that I was about to lose consciousness. A few inhalations of spirits of ammonia, however, gave me the necessary stimulation to prevent a loss of consciousness, and I was not again troubled with faintness.

At this time I noticed that the sense of great weight in the legs had entirely disappeared and that numbness was now only distinguishable in the toes. The suturing of the deeper tissues was exceedingly painful, and it seemed to me that the anesthesia had entirely passed off in this region of the body. Before the deep sutures were all in place the numbness had entirely passed from the toes, as was proved by the fact that when moved against each other the sensation appeared to be entirely normal. At this period my courage for a moment failed, and I begged for a few inhalations of chloroform, but was assured that the operation would be completed in five minutes, so took a new grip upon myself and endured to the end.

The second operation consumed just twenty minutes. There were no complications encountered in this case, and as I was suffering so acutely, every one connected with the operation worked as rapidly as possible, that they might the sooner bring my suffering to an end.

The whole surgical procedure consumed just fifty-five minutes, three for the anesthesia and fifty for the operations. At no time while under the anesthetic effect of the drug was there any acceleration or diminution of the heart action or respiration; neither was there any stimulation nor depression of the mental faculties. The headache so frequently complained of for several days or weeks did not occur in my case. The only unpleasant after effects experienced from the operations was the accumulation of gas in the stomach and bowels, that so frequently follows abdominal operations and which is in no way related to the kind of anesthetic employed.

From this experience with spinal anesthesia I am led to the following conclusions:

First.—That spinal anesthesia is a safe and reliable method of producing insensibility to pain in certain surgical operations in normal individuals.

Second.—From the fact that this method used with the usual dose does not seem in any way to disturb the cardiac or respitory nerve centers, it would be indicated in those cases in which chloroform and ether would be contraindicated.

Third.—That for all operations below the diaphragm, which do not consume more than thirty minutes for their completion, it is a most admirable method of anesthesia.

Fourth.—In operations above the diaphragm it would seem to be of doubtful utility with the dose usually employed. Morton is in the habit of making the injection with considerable force and elevating the lower extremities when using this method if the operation is to be upon the upper portion of the body, and he claims to be successful. In the case referred to in the body of this article as having fracture of the mandible, Morton's method did not give the desired result. It is possible, however, that by increasing the dose, anesthesia of the head might be successfully secured, but as I have had no experience with a larger dose than one grain, I would not hazard an opinion upon it.

Fifth.—It would not be wise to use this method of anesthesia in the case of patients who have a great nervous dread of surgical operations and in whom the consciousness of being operated upon might produce mental shock, or in the case of patients who could not be controlled by reason. —*Items of Interest.*

